# Molecular Mechanisms of Immunosenescene and Inflammaging: Relevance to the Immunopathogenesis and Treatment of Multiple Sclerosis

**DOI:** 10.3389/fneur.2021.811518

**Published:** 2022-02-25

**Authors:** Océane Perdaens, Vincent van Pesch

**Affiliations:** ^1^Laboratory of Neurochemistry, Institute of Neuroscience, Université catholique de Louvain (UCLouvain), Brussels, Belgium; ^2^Department of Neurology, Cliniques universitaires Saint-Luc, Université catholique de Louvain (UCLouvain), Brussels, Belgium

**Keywords:** multiple sclerosis, immunosenescence, inflammaging, T/B cells, oligodendrocytes, microglia, astrocytes, disease modifying therapies

## Abstract

Aging is characterized, amongst other features, by a complex process of cellular senescence involving both innate and adaptive immunity, called immunosenescence and associated to inflammaging, a low-grade chronic inflammation. Both processes fuel each other and partially explain increasing incidence of cancers, infections, age-related autoimmunity, and vascular disease as well as a reduced response to vaccination. Multiple sclerosis (MS) is a lifelong disease, for which considerable progress in disease-modifying therapies (DMTs) and management has improved long-term survival. However, disability progression, increasing with age and disease duration, remains. Neurologists are now involved in caring for elderly MS patients, with increasing comorbidities. Aging of the immune system therefore has relevant implications for MS pathogenesis, response to DMTs and the risks mediated by these treatments. We propose to review current evidence regarding markers and molecular mechanisms of immunosenescence and their relevance to understanding MS pathogenesis. We will focus on age-related changes in the innate and adaptive immune system in MS and other auto-immune diseases, such as systemic lupus erythematosus and rheumatoid arthritis. The consequences of these immune changes on MS pathology, in interaction with the intrinsic aging process of central nervous system resident cells will be discussed. Finally, the impact of immunosenescence on disease evolution and on the safety and efficacy of current DMTs will be presented.

## Introduction

With the aging of the world population, seniors aged over 65 years, that account for 9.3% of the global population in 2020, are predicted to have doubled in absolute number by 2050, representing 15.9% (1). This increase in life expectancy inevitably has an impact on disease prevalence and incidence, especially of chronic diseases. Health care systems worldwide must face this demographic evolution within the next decades. Biological aging is the decline in homeostasis, with functional alterations of all organs and tissues, resulting in an increase of morbidity and mortality (2). On a cellular level, senescent cells, accumulating with age, are arrested in their cell cycle, but are still active, although functionally dysregulated and affecting their microenvironment by secreting soluble signaling factors (interleukins, chemokines, growth factors), proteases, or insoluble proteins/extracellular components. These constitute the so-called senescence-associated secretory phenotype (SASP) exerting a paracrine pro-inflammatory effect (3, 4). The immune system, which is continuously operating throughout life, is prone to these age-related changes, referred to as immunosenescence (5). Immunosenescence affects both the innate, and, to a greater extent, the adaptive immunity. It is postulated to explain increased prevalence of infections, cancers and auto-immune diseases and reduced response to vaccination in the elderly (6). On the contrary, the purpose of cell cycle arrest in senescent cells is to prevent cellular escape into tumoral processes (7). However, no single immune change associated with senescence explains health-related consequences of aging. Hence, a longitudinal study proposes an age-related ‘immune risk phenotype' associated with poorer survival, characterized by an inversion in the CD4^+^/CD8^+^ T cell ratio, the expansion of the terminally differentiated CD8^+^CD28^−^ T cells, lower B cell numbers and seroconversion for cytomegalovirus (CMV) (8–10). Moreover, a chronic, sterile low-grade inflammation occurs concurrently, named inflammaging, mutually interacting with immunosenescence. Continuous antigenic load and stressors stimulate the innate immune system, mainly macrophages, to produce pro-inflammatory cytokines, such as interleukin (IL) 1, IL6, or tumor necrosis factor (TNF), also part of the SASP (11). However, centenarians aging healthily have an inverted immune risk phenotype and a heightened inflammaging profile properly counterbalanced by anti-inflammaging (12–14). Hence, Franceschi et al. argument that diseases arise when this equilibrium is broken (11, 15).

The multiple sclerosis (MS) population older than 65 years is increasing worldwide due to improving life expectancy with MS, although the latter remains 6–10 years shorter as compared to the general population (16, 17). There is growing awareness about the implications of aging with MS, due to immunosenescence, the high burden of comorbidities and the lack of knowledge on long-term effects of exposure to disease modifying therapies (DMTs). The safety, efficacy and benefit of DMTs in this population are unknown, since patients over 55-to-60-years-old are generally excluded from clinical trials (17). Furthermore, while remitting-relapsing MS (RRMS) is the prominent phenotype in younger patients, older patients more likely have primary or secondary progressive MS (PPMS/SPMS), in which chronic inflammation and neurodegeneration, due to failure in myelin repair and axonal loss, is considered to prevail (18). However, the pathophysiology underlying the progression of the disease with aging remains incompletely understood.

We aim to review and compare current knowledge on immunosenescence and inflammaging, relative to MS and other autoimmune diseases (AIDs), such as systemic lupus erythematosus (SLE) and rheumatoid arthritis (RA), as these AIDs are amongst the most studied in this context (19–21). In the setting of MS, we will also focus on the concomitant senescence of central nervous system (CNS) resident cells in order to answer several questions. (a) What is the evidence or the lack thereof to consider MS a disease of premature immunosenescence? (b) What are the common or different immunosenescence features found in MS and other autoimmune diseases? (c) Are epigenetic changes involved in immunosenescence? (d) Is the age-related evolution of MS toward a progressive phenotype linked to immunosenescence? (e) Does immunosenescence expose aging MS patients to increased risks of infection and cancer, especially when taking DMTs?

## General Mechanisms of Immunosenescence

Immunosenescence is defined as the physiological aging of the immune system (22). Immune cells are generated from hematopoietic stem cells (HSCs) throughout life and differentiate stepwise, undergoing selection and proliferation pressure upon antigenic contact. They are thus especially prone to senescent processes. Changes related to immunosenescence are more preeminent within the adaptive than the innate immune system [reviewed by (23, 24)].

Cell cycle arrest of aging cells is initially a protective phenomenon against increasing cellular damage and tumorigenesis (7). The pro-inflammatory SASP (IL6, IL8, matrix metalloproteinase (MMP) 1/3, monocyte chemotactic protein (MCP) 2/3, insulin growth factor binding proteins) of senescent cells constitutes a removal-signal directed toward immune cells (4). However, due to age-related dysfunctions in the immune system, this clearance partly fails (25). The pro-inflammatory and oxidative context occurring during the aging process, enhances the nuclear factor kappa-light-chain-enhancer of activated B cells (NF-kB) pathway, a key regulator of inflammation (26). This compensatory mechanism may become self-deleterious, since cumulative cell debris, self-antigens and the inflammatory SASP contribute together to inflammaging, altering cell, tissue, organ and organism homeostasis [reviewed by (23)].

Immunosenescence has been implicated in reduced defenses against infections and reduced response to vaccination (due to a reduced antigenic response by T and B cells), an increased risk of cancer (due to an imbalance between the function of regulatory cells and cytotoxic CD8^+^ T cells) and auto-immune diseases (due to reduced clearance of apoptotic cells and reduced antibody diversity, with however an increased susceptibility to molecular mimicry) (27–30). These risks might be counterbalanced by the subject's intrinsic (e.g., genetic polymorphisms, epigenetics) and extrinsic factors (e.g., the individual's history of past immune reactions, referred to as immunobiography, as well as environmental factors) (11, 15).

### T Cells

With aging, the pool of naïve T cells is reduced due to thymic involution and reduced bone marrow proliferative capacity. Both the thymus and the bone marrow lose their epithelial/stromal cell frame, which is replaced by adipocytes, resulting in a reduction in HSC proliferation (31, 32). Moreover, a general shift of HSCs from the lymphoid to the myeloid lineage is observed. Thymic T cell output, measurable by T cell receptor (TCR) excision circles (TRECs), is reduced with age. TRECs are stable extrachromosomal DNA byproducts resulting from thymic TCR rearrangements. TRECs are not replicated and are therefore diluted with cell division (33).

Homeostatic proliferation, driven by dendritic and B cells upon exposure to IL7 and IL15, occurs initially to compensate for the reduced peripheral input of naïve T cells, but results in the clonal expansion of memory T cells and a depleted TCR repertoire (34–37). The proportion of T helper (Th) cells decreases due to defective antigen presentation and an impaired TCR response, resulting in a reduction of TCR-mediated proliferation (38). The reduced expression of CD40 ligand (CD40L) on CD4^+^ T cells impairs their binding to B cells and thus their ability to function as T helper cells (39).

With aging, a shift from Th1 to Th2 cells is observed, due to decreased IL2 production, although this is disputed (40, 41), while the percentage of Th17 cells is increased in subjects aged over 65 as compared to younger subjects (42). Moreover, memory T cells are resistant to apoptosis, hence reenforcing their numerical increase (43).

Overall, during senescence, the number of CD4^+^ T cells decreases and CD8^+^ T cells increases resulting in an inverted CD4^+^/CD8^+^ ratio (<1) (10). Antigen-experienced T cells proliferate and differentiate into terminally differentiated memory cells with shortened telomeres that eventually lose CD28 expression, a costimulatory signal involved in T cell activation and survival (44). This loss, mainly observed in memory CD8^+^ T cells, has been linked to aging and immunosenescence, and is partly enhanced by chronic antigenic stimulation, especially by CMV, with a ten-fold factor for CD4^+^ and 2-fold for CD8^+^ T cells (45, 46). These CD28^−^ cells express the natural killer (NK) receptor NKG2D which provides an antigen-independent activation signal (along with the NK adaptor molecule DAP12), bypassing the missing costimulatory signal CD28, and enhancing their autoreactivity (47, 48). Moreover, these cells express cytokines [interferon (IFN)g, TNFa] and cytotoxic molecules (granzyme A/B, perforin) upon expression of the eomesodermin factor, and are resistant to apoptosis [by expressing B cell lymphoma 2 (BCL2) and Fas-associated death domain-like IL-1-converting enzyme inhibitory protein (FLIP)] (49–51). Finally, they express chemokine receptors [e.g., C-X3-C Motif Chemokine Receptor 1 (CX3CR1)], which might favor their migration to inflammation sites (51, 52).

In summary, immunosenescence in T cells is characterized by a physiologically reduced pool of naïve T cells and an increase in memory, particularly CD8^+^, T cells, that have lost CD28 and express NKG2D, the first increasing T cell self-reactivity in secondary lymphoid organs, the second reducing their threshold for antigen-specific activation hence enabling an antigen-independent activation. The autoreactivity of senescent T cells is enhanced by the clonal expansion of memory T cells and the reduced TCR repertoire. These changes can also partly explain the reduced immune defenses against new pathogens observed during aging, as senescent cells are considered functionally deficient, contrary to exhausted T cells, which are considered dormant and can still respond to a previously encountered antigen (15).

### B Cells

B cell numbers, phenotypes and functions change with age [reviewed by (53, 54)]. Reduced B cell output is attributed to global changes in hematopoiesis, as described above. Moreover, peripheral B cell survival factor levels, such as B cell activating factor (BAFF) and A proliferation-inducing ligand (APRIL) are reduced in the elderly (55). In addition, stromal cell-derived IL7 production is reduced, whereas the increased pro-inflammatory cytokine levels [TNFa, IL1b, and transforming growth factor (TGF)b] withhold the B progenitor cells from the IL7-rich niches, hence impairing B lymphopoiesis and reducing the pro-B cell immunoglobulin (Ig) heavy chain V-DJ rearrangement and thus the pre-B cell receptor (BCR) repertoire (56–59). As a consequence, absolute and relative numbers of peripheral CD19^+^ B cells are reduced, while the proportions of B subsets remain stable with age in humans (53).

With aging, naïve mature (IgD^+^CD27^−^) B cells decrease while exhausted double negative memory (IgD^−^CD27^−^) B cells increase. IgM unswitched (IgD^+^CD27^+^) and switched (IgD^−^CD27^+^) memory B cells remain generally stable (53, 54). The immature transitional immunoregulatory CD24^high^CD38^high^ B cell subset decreases with age, so does its IL10 production (60).

The humoral immune response is altered during senescence, since antibodies are reduced not in quantity but in their diversity and affinity and show cross-reactivity to self- and foreign antigens. This is due to a decrease in antibody class switch and affinity maturation in clonally expanding B cells, related to the downregulation of the E47 transcription factor and activation-induced deaminase (61, 62). This alters the ability to mount a rapid secondary antibody response. Furthermore, a progressive decline in germinal center formation during aging decreases somatic hypermutation, *i.a*. in IgD^−^CD27^+^ B cells and even more in double negative B cells (63). Moreover, immunosenescent B cells lack the support of the Th cells, since Th cells are reduced in number, express less CD40L, and are less exposed to antigen presentation by antigen presenting cells (APCs), due to a reduced expression of the major histocompatibility complex of class II (MHC-II) on the latter (39, 64).

Interestingly, double negative memory B cells express chemokine receptors, C-X-C Motif Chemokine Receptor 3 (CXCR3), although reduced with age, C-C Motif Chemokine Receptor (CCR)6 and CCR7, and are thus prone to migrate to the inflammation sites (65). Moreover, these cells are pre-activated and can produce pro-inflammatory cytokines, and granzyme (66, 67). They undergo an antigen-driven BCR hypermutation.

Finally, low but steadily expanding CD11b^+^CD11c^+^CD21^−^ age-associated B cells (ABCs) have been identified in the elderly, in response to antigenic stimulation, and linked to autoreactivity. This functionally exhausted memory subset is driven by the T-box transcription factor (TBET) and is activated synergically upon stimulation of the BCR and Toll-like receptors (TLR)7 and 9. ABCs produce pro-inflammatory cytokines (e.g., TNFa), inhibit B lymphopoiesis and favor Th17 polarization (68–70). They possibly derive from follicular B cells and exhibit downregulation of Epstein Barr Virus (EBV) receptor CD21 due to chronic EBV stimulation.

In summary, the changes in B cell phenotypes, and recirculation, along with their altered humoral response contribute to immunosenescence and can explain the reduced response to vaccination and increased susceptibility to infections, while the clonal expansion of B cells cross-reactive to self-antigens can favor autoimmunity.

### Immunosuppressive/Regulatory Cells

Natural, thymic-derived CD4^+^CD25^+^FOXP3^+^ regulatory T cells [(n)Tregs], mainly with an effector memory phenotype (CD45RO^+^/CD45RA^−^), increase with age in human in relative and absolute numbers, so does the expression of their transcription factor, forkhead box P3 (FOXP3), possibly due to their better survival in the periphery, since they reduce the expression of the pro-apoptic BCL2 interacting mediator of cell death (BIM) (71–74). Functionally, CD4^+^ Tregs of aged humans and mice can suppress CD4^+^ and CD8^+^ T cell proliferation and IFNg production, but in aged mice they could not suppress IL17 production (75, 76).

Likewise, natural CD8^+^FOXP3^+^ nTregs increase with age, while their peripheral inducible capacity is reduced (77, 78). Functionally CD8^+^ nTregs retain the same suppressive ability independently of aging. Interestingly, a CD8^+^CD28^−^FOXP3^+^ cell subset has been described, in agreement with the overall increase of CD8^+^CD28^−^ T cells (79). Finally regulatory B cells and myeloid-derived suppressor cells also appear increased with age but have been less studied (80).

In summary, Tregs participate to the immunosenescent process by their increased number and their safeguarded suppressive activity, except against Th17 cells [reviewed by (79, 81)]. This correlates with increased cancer incidence, since Tregs suppress the CD8^+^ T cell anti-tumor response, and with an increased risk of infection and viral reactivation, since they suppress the anti-pathogen response (72, 82, 83). They have also been linked to neurodegeneration due to their differential interaction with microglia both in the presence and absence of effector T cells (84).

### Innate Immunity

Although less affected by immunosenescence, partly because HSCs are redirected toward the myeloid lineage, innate immunity still displays mainly functional changes [reviewed by (85)].

With aging, dendritic cells show less migration abilities, less responsiveness to TLR stimulation, reduced pathogen processing (phagocytosis, endocytosis) and antigen presentation. This is attributed to mitochondrial dysfunction, resulting in the production of reactive oxygen species (ROS) (86, 87). These alterations affect T cell stimulation and consequently the CD8^+^ T cell cytotoxic response. Type I (IFN-I, i.e., IFNa/b) and III (IFN-lambda) IFN production is decreased, but they still produce IL6 and TNFa (87, 88).

Several important functions of neutrophils are reduced with aging: chemotaxis, phagocytosis, production of ROS and neutrophil extracellular traps (NET). Opsonization of antibody-bound pathogens is dwindled (89, 90).

Monocytes shift from the classical (CD14^++^CD16^−^) to the pro-inflammatory non-classical (CD14^+^CD16^++^) phenotype, however with some discrepancies on their expression of Human Leukocyte Antigen-DR isotype (HLA-DR) and CX3CR1 (91–93).

Macrophages also produce less ROS, IL6, and TNFa. They display impaired phagocytosis resulting in reduced antiviral response and impaired auto-/mitophagy, resulting in the accumulation of altered organelles and molecules (25, 94). Moreover, they express less TLRs and MHC-II on their surfaces, thus impairing their ability to present antigens to CD4^+^ T cells (95, 96).

The slight net increase in the total number of NK cells is in fact due to a decrease of the immunoregulatory CD56^bright^ and an increase of the cytotoxic CD56^dim^ NK cell subsets. These show however impaired degranulation and thus decreased cytotoxic abilities on a per cell basis. IL2/12-mediated secretion of immunomodulatory cytokines (e.g., IFNg) and chemokines is reduced, while production of IL1/4/6/8 and TNFa is increased (97, 98). Furthermore, the central maturation of NK cells is incomplete (99).

### Epigenetics and Telomeres

Insight into the function of microRNAs (miRNAs) has rapidly grown over the past two decades. miRNAs are small non-coding RNAs regulating gene expression post-transcriptionally by binding to their target messenger RNA (mRNA) and mostly inhibiting its translation (100). Overall, miRNA transcription decreases with age [e.g., miR-17/92a/181a in peripheral blood mononuclear cells (PBMCs)] (101, 102), and they have been linked to several mechanisms underlying cellular senescence [reviewed by (103)]. Oxidative stress can affect positively or negatively miRNA expression (104–107). On the opposite, the downregulation of miR-146a enhances NADPH oxidase (NOX) as it targets its subunit *NOX4* (108). The downregulation of the miR-17-92 cluster and the upregulation of miR-210 enhance ROS production (109, 110). Furthermore, miR-210 induces senescence-associated heterochromatin loci and double-strand DNA breaks and is involved in mitochondrial dysfunction by targeting a subunit of the electron transport chain (110, 111). miR-34a and -101 inhibit autophagy (112, 113). By targeting the pro-proliferative cyclin A2, an antagonist of p21, miR-29a and -124, both induced by p53, enhance p21 expression, and thus senescence by cell cycle arrest (114). miR-20a is also an indirect p53-senescence inducer (115). Furthermore, the miR-17-92 cluster and miR-106b family target *p21*, while p53 inhibits the miR-17-92 cluster (107, 116–119). Finally, miR-9/96/145 were upregulated concomitantly to the downregulation of insulin growth factor 1 receptor (a miR-96/182-target) and forehead box protein O1 (FOXO1, a miR-145/132-target) in the PBMCs of elderly subjects, but miR-132 and -182 were not differentially expressed in this study (120).

Telomeres [reviewed by (121)] are repetitive hexameric sequences (TTAGGG) at the chromosome end of 10–15 kb at birth that shorten by 40–200 bp with each cell division, although length of shortening per mitosis might vary as it is higher in memory than in naïve cells. Critically short telomeres induce a signal for p53-dependent cell cycle arrest. Telomerase is a ribonucleoprotein complex comprising a catalytic subunit, telomerase reverse transcriptase (TERT), which can elongate the hexameric sequences. Telomere length depends on the balance between telomere shortening and telomerase activity, but overall decreases with age. Telomerase activity is increased in stem cells, but also in lymphocytes, where it is the highest in the germinal center (122, 123). However, this activity in the latter is not enough to slow down telomere shortening. Oxidative stress and an increased replication rate upon repetitive stimulation during inflammation, progressively reduce telomerase activity, paralleling the loss of CD28, and hastens telomere attrition and thus cellular senescence (121, 124, 125). Remarkably, centenarians have longer telomeres with lower levels of basal inflammation (14). Interestingly, miRNAs can induce telomere dysfunction and cellular senescence, as miR-138 and -512-5p inhibit *TERT*. miR-155 targets telomeric repeat-binding factor *(TRF)1*, miR-23a targets *TRF2*, which both ensure telomere maintenance (126–129).

Several miRNAs are considered as major immuno-microRNAs, playing a role in immune cell homeostasis and senescence, but also in inflammatory responses and inflammaging [reviewed by (130, 131)]. Age-dependent changes in miRNAs diverge between naïve, central and effector memory CD8^+^ T cells, but miR-181a is commonly downregulated in the aged cells of all 3 subsets. The most changes are uncovered in the naïve T cell subset, where they are correlated with the decline of FOXO1 signaling, evidenced by the downregulation of IL7 receptor and *CCR7*, and the alteration of TNFa and NF-kB signaling (132). The SASP in senescent cells induces the delayed expression of miR-146a/b to target IL1 receptor associated kinase (*IRAK*)*1* and to compensate downstream NF-kB-dependent inflammation mediated by IL6/IL8 (133). miR-223 downregulates the NF-kB pathway and the inflammasome NLRP3 (134). Contrarily, NF-kB induces miR-155, which inhibits suppressor of cytokine signaling *(SOCS)1*, allowing T effector expansion and T memory formation and maintenance (135). miR-17/19b/20a/106a were downregulated in CD28^−^ vs. CD28^+^ and in CD28^+^ T cells of old vs. young donors alongside the upregulation of *p21* (109). The miR-17-92 cluster and miR-21 support the differentiation into T effector cells (136, 137). On the contrary, the T effector response upon viral infection is delayed in miR-155- or miR-181a-deficient (CD8^+^) T cells, and cells differentiate to central rather than effector memory cells (138, 139). Moreover, with age, the decrease of Yin-Yang 1 and T cell factor 1 results in the downregulation of miR-181a, which induces dual specific phosphatase *(DUSP)6* expression. The latter impairs extracellular signal-regulated kinase (ERK)-dependent TCR sensitivity (140). Furthermore, miRNAs can impair B cell differentiation in the elderly. miR-155, that targets activation-induced deaminase, and miR-16, that targets *E47*, are increased in memory B cells and even more in double negative B cells (141, 142).

Epigenetics translate the effect of the environment on gene expression [reviewed by (143)]. While methylation, catalyzed by DNA methyltransferases (DNMT), can vary at a cell-base level, the global methylation rate is reduced with age, possibly by passive demethylation and reduced activity of DNMT1 (144). Hypomethylation allows gene expression. Naïve CD4^+^ T cells display age-associated hypomethylation sites in immune-related pathways [TCR signaling, Fc gamma receptor (FCgR)-mediated phagocytosis, mammalian target of rapamycin (mTOR) and insulin signaling, antigen processing and presentation], while hypermethylation was observed in cell proliferation pathways [Wnt and mitogen-activated protein kinase (MAPK) signaling] (145, 146). Interestingly, centenarians display a slower reduction of DNA methylation level (147). Sirtuins (SIRT) encode NAD^+^-dependent histone deacetylases (HDAC) and maintain the genome's integrity during cellular stress. Downregulation of *SIRT1* and *SIRT3* in PBMCs of healthy elderly subjects was accompanied by the upregulation of miR-9, which targets *SIRT1*, and miR-34a (148). Oxidative stress induces miR-195, that targets *SIRT1*, which is associated with reduced telomerase activity (149).

In summary, telomeres shorten with age due to cell replication and oxidative stress, despite sustained telomerase activity. Epigenetically, global hypomethylation occurs with aging. Interestingly, miRNAs are involved in cellular senescence through different mechanisms [oxidative stress, mitochondrial dysfunction, cell cycle arrest (p53 pathway), telomere attrition, and inflammation].

## Immunosenescence in Auto-Immune Diseases

Immunosenescence is associated with an increase in the incidence of several AIDs (150). Some diseases show a bimodal age of onset (e.g., RRMS vs. PPMS, MOG-antibody diseases), others almost exclusively occur in the elderly (e.g., polymyalgia rheumatica, giant cell arteritis) (30, 151–153). A German study showed an age-decreasing female incidence of SLE peaking at the age of 20–25 and to a lesser extent at menopausal age, and an age-increasing male incidence peaking at the age of 65–70 (154). RA-incidence and prevalence increase with age to peak at age 50–54 and 60–64, respectively (155). Serum autoantibody titers are generally higher in elderly subjects, even without overt AID. Moreover, the binding of circulating antibodies to random peptides, especially with a di-serine motif, increases with age (150, 156).

Interestingly, AIDs show inflammaging and features of immunosenescence at an earlier age. Age-associated defects at the cellular level, classified under the nine common denominators of aging (2), and the resulting impaired immune function create an unstable state, that may predispose for tolerance failure and occurrence of autoimmunity (152, 157). Herein, we will focus on the effect of stem cell exhaustion, altered intercellular communication (e.g., by inflammaging), proteostasis (i.e., the maintenance of a functional cellular protein pool) loss, telomere attrition, genomic instability, mitochondrial dysfunction, and epigenetic alterations in MS, SLE, and RA.

Lymphopenia-induced homeostatic proliferation leads to clonal expansion and TCR repertoire contraction over time. Furthermore, priming by cytokines produced during inflammaging, can transiently reduce the TCR stimulation threshold (by ERK phosphorylation), consequently interfering with tolerance maintenance and promoting autoreactive T cells [reviewed by (152)]. TNFa engages cellular senescence by inducing interferon response genes, cytokine secretion, and ROS production (158). Moreover, the Th17/Treg imbalance contributes to trigger autoimmune diseases (42).

Three cell types are unique to immunosenescence, i.e., CD28^−^ T cells, linked to cytotoxicity, double negative B cells and ABCs, linked to autoantibody production, and might play a role in autoimmunity (48, 66, 68). The CD4^+^CD28^−^ cell population is enlarged in subjects with autoimmune diseases compared to age-matched controls, with the highest percentage in RA, followed by RRMS and SPMS and finally SLE, and is positively correlated with age and CMV seropositivity. These cells are enriched in granzyme A and B, and perforin and their TCR repertoire is contracted as compared to CD28^+^ T cells. The latter appears even stronger in MS/RA than in healthy controls (159).

Cells recycle long-lived proteins, damaged organelles, and aggregates by autophagy via the lysosomes, for the synthesis of new proteins or for energy production, thus ensuring cellular homeostasis, especially under nutrient-/energy-poor conditions (160, 161). Autophagy declines with age, as seen by the downregulation of autophagy-related protein (ATG)5, ATG7 in the human aged brain (162, 163). Interestingly, autophagy and inflammation can reciprocally induce and suppress each other. Autophagy is induced by TLRs but inhibited by Th2 cytokines. Conversely, it blocks the inflammasome, and thus the IL1b response. It prevents ROS production by degrading dysfunctional mitochondria, but it also promotes the survival and differentiation of immune cells [reviewed by (164)].

In AIDs, metabolic reprogramming for energy production may fail leading to hyperreactive immune cells and an increase in oxidative stress. Oxidative stress and mitochondrial dysfunction contribute to (immuno-)senescence and inflammation through decreased redox capacity (glutathione depletion), activation/oxidation-induced cell apoptosis (with defective clearance and release of the cell content inducing TLR), mitochondrial DNA (mtDNA) damage, defective bioenergetics (ATP depletion) and production of neoantigens (165, 166). Moreover, oxidative stress, inflammation, and increased leukocyte renewal accelerate telomere shortening [reviewed by (166)].

### Immunosenescence in Multiple Sclerosis

**Figure 1:**
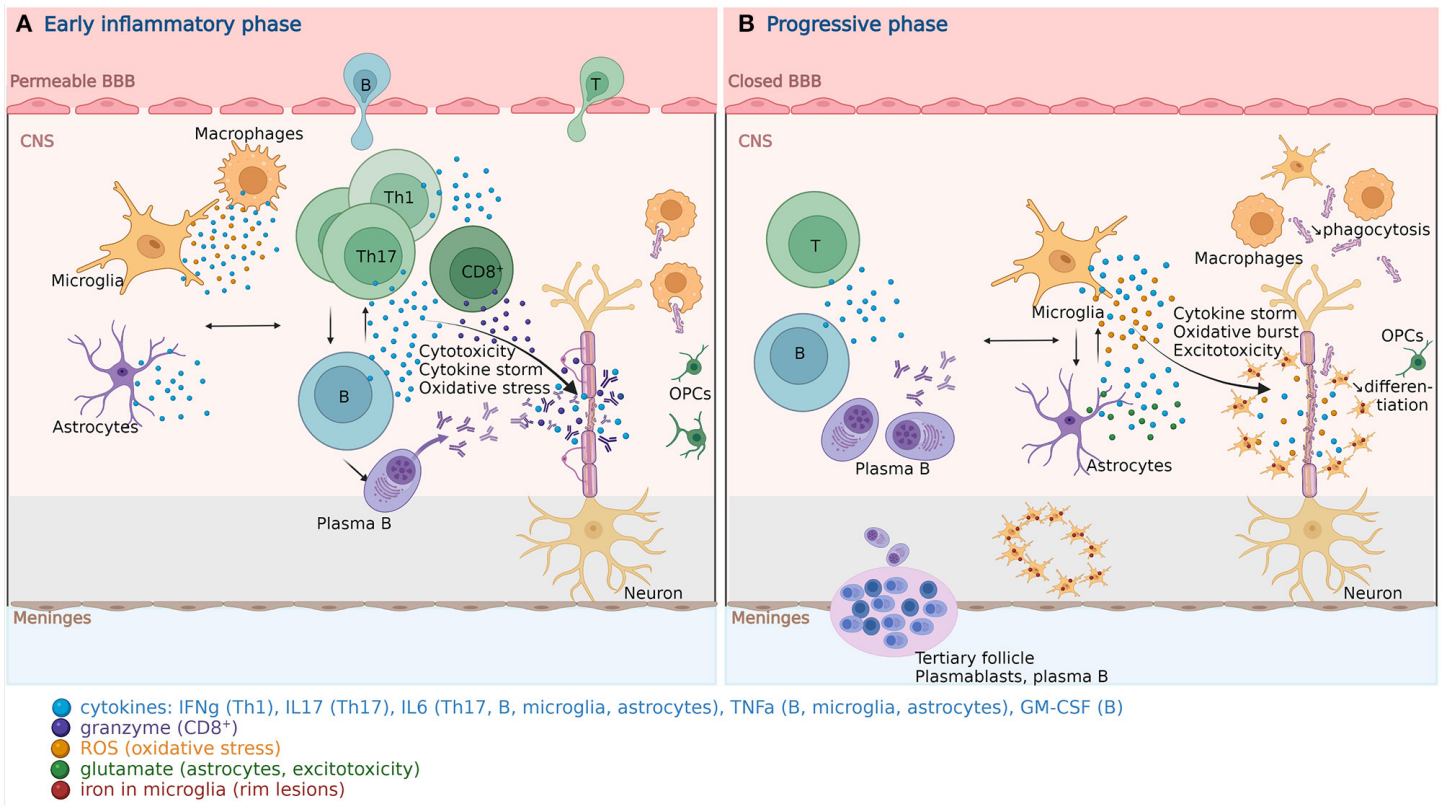
Pathophysiology of multiple sclerosis. **(A)** In the early inflammatory phase of MS, peripheral adaptive immune cells infiltrate the CNS through a disrupted BBB. These activated cells interact with each other and the resident cells of the CNS. They secrete cytokines (e.g., IFNg by Th1, IL6/17 by Th17, GM-CSF, IL6, TNFa by B cells) and cytotoxic molecules (e.g., granzyme B by CD8^+^ T cells). B cells can further evolve into autoantibody-producing plasma cells. As a consequence, T and B cells activate macrophages and microglia, which produce cytokines, nitric oxide, and ROS. This cytotoxic pro-inflammatory environment breaks down the myelin sheaths around axons and induces energy failure in the axon. Macrophages and microglia can still clear the myelin debris, allowing for the recruitment of OPCs that will partially remyelinate the lesion. **(B)** In the progressive phase of MS, T and B cell infiltrates are reduced. Remarkably, plasmablasts and plasma B cells form tertiary follicle-like structures in the meninges. The BBB is closed and the inflammation is sustained by innate CNS-resident cells, i.e., microglia and astrocytes. They produce cytokines (TNFa, IL6) and release ROS, causing myelin damage. Although microglia are primed into a pro-inflammatory phenotype, their phagocytic capacities are reduced. These features also characterize the senescence-associated phenotype of microglia and astrocytes. Myelin debris are improperly cleared, OPCs are less recruited and fail to differentiate. TNFa-mediated glutamate release from astrocytes results in excitotoxicity causing axonal damage. The ferrous iron released from the myelin, where it accumulates with age, is oxidized, which produces ROS, and incorporated by microglia, forming a phenotypical rim around the demyelinating lesions, both in the white and gray matter. These successive events are self-sustained and enhanced by senescent processes, resulting in a major oxidative burst, causing mitochondrial dysfunction, mitochondrial DNA damage, energy failure and axonal loss. BBB, blood brain barrier; B, B cell; CNS, central nervous system; GM-CSF, granulocyte-macrophage colony-stimulating factor; IFN, interferon; IL, interleukin; MS, multiple sclerosis; OPC, oligodendrocyte progenitor cell; ROS, reactive oxygen species; T, T cell; Th, T helper cell; TNF, tumor necrosis factor. Created with BioRender.com.

In MS, activated peripheral autoreactive CD4^+^ T cells, migrate through a disrupted blood brain barrier (BBB) into the CNS. They are reactivated upon antigenic contact, and interact with other peripheral immune cells (CD8^+^ T and B lymphocytes, monocytes, and macrophages). They activate microglial cells and astrocytes to induce demyelination, oligodendrocyte apoptosis and axonal damage (Figure 1A) (18). MS patients present at a younger age with some features of immunosenescence seen in aged healthy controls, suggesting that it is possibly involved in MS pathogenesis [reviewed by (19, 167)].

#### Innate Immunity

Circulating neutrophils in RRMS patients produce more inflammatory markers and NETosis, and are resistant to apoptosis. The serum/plasma levels of neutrophil-activating chemokines and neutrophil-derived enzymes [e.g., C-X-C motif ligand (CXCL)1, CXCL8, elastase] are positively correlated with new inflammatory lesions. Neutrophils are also found in the cerebrospinal fluid (CSF) at onset and early in relapse, but decrease with disease duration (168–170). Regarding monocytes, discrepancies exist due to study population and staining strategy differences. Some describe an increase in classical and non-classical monocytes in inactive RRMS as compared to progressive MS, while others state an increase in non-classical monocytes in a mixed MS population (171, 172). The beneficial or detrimental role of NK cells is still debated [reviewed by (173)]. The peripheral blood of PP/SPMS counts more CD56^dim^ NK cells, while the CD56^bright^ NK cells are expanded in the CSF of untreated RRMS patients due to their higher migratory capacity, which might counterbalance the CNS inflammation (174, 175). However, their immunoregulatory and cytolytic functions appear to be impaired (176, 177). Moreover, NK cells in the CNS could delay remyelination, as they suppress the reparative properties of neural stem cells in experimental autoimmune encephalitis (EAE) (178).

#### T Cells

The bone marrow cellularity is reduced and the *in vitro* proliferative capacity of mesenchymal stromal cells, supportive of hematopoiesis, declines with age, even more in PP/SPMS, while CD34^+^ HSC numbers remain stable in MS but the frequency of colony forming cells is low (179, 180). Moreover, thymic involution is accelerated in MS, given that TREC levels are, at all ages lower than in age-matched healthy controls and progressively decrease with age (159). Hence, the CD8^+^ naïve T cell pool is reduced, while data regarding the effect of age on naïve CD4^+^ T cells in MS are more discrepant (181–183). Interestingly, the TCR repertoire is more diversified in MS (184). Moreover, Th17 cells are largely involved in MS and increased in the periphery (185, 186). The inverted CD4^+^/CD8^+^ ratio, the hallmark of the immune risk phenotype, mostly does not apply in MS (187–189). This ratio was however decreased in the CSF of patients on natalizumab treatment (190, 191).

Both effector memory CD4^+^ and CD8^+^ T cells may enhance the chronic inflammatory responses to neuroantigens in MS and EAE (192, 193). Notably, inoculation with CD4^+^ memory rather than effector T cells in EAE preferentially induced marked CNS inflammation (194, 195). Herein, memory CD4^+^ T cells are increased in the blood and the CSF during active disease (196). Central and effector memory CD8^+^ T cells are increased, independently of disease activity, in the blood, and the latter also in the CSF, and the CNS tissue (192, 196–198).

The cytotoxic CD4^+^CD28^−^ population is enriched with advancing age in RR/SP/PPMS, while it remains stable in healthy controls, and has been linked to disease severity in EAE and MS (159, 199–201). They are partly autoreactive to myelin basic protein (MBP) (199). Since these cells express CX3CR1, they might infiltrate the CNS where the CSF levels of its ligand fractalkine were found elevated in MS (52).

#### B Cells

B cells play a central role in MS development and progression [reviewed by (202)]. Antigen-driven clonally expanded B cells produce pro-inflammatory cytokines [TNF, lymphotoxin (LT)a, IL6, granulocyte-macrophage colony-stimulating factor (GM-CSF)] and chemokines through the NF-kB pathway. Memory B cells act as APCs, and hereby prompt the proliferation and activation of T and myeloid cells. B cells, stimulated by Th follicular cells, differentiate into immunoglobulin-producing plasmablasts and plasma cells that accumulate to eventually form tertiary follicle-like structures in the leptomeninges during disease progression and are notably involved in inducing subpial demyelination.

Transitional B cells (CD24^high^CD38^high^) are reduced in the blood and are functionally defective in RRMS (produce less IL10). They have been found in the CSF while they were absent in CSF samples of other inflammatory neurological diseases (203, 204). The proportions of peripheral naïve B cells decrease and memory B cells increase with age in controls (54), but remain stable in MS, except during relapse. Interestingly, the proportion of CSF class-switched memory B cells is increased in adult MS whereas the relative numbers of unswitched memory B cells are increased in pediatric MS (205). In B cells from MS patients, a preferential naïve-to-memory transition possibly occurs as the production of LTa and TNFa by memory CD27^+^ B cells was high and comparable to that of healthy controls, whereas the production of IL10, normally expressed by naïve CD27^−^ B cells was reduced (206).

Double negative (IgD^−^CD27^−^) B cells and ABCs (CD11c^+^CD21^−^ or CD21^low^) are increased in a proportion of MS patients before the age of 60 years, whereas they are mainly found above 60 in healthy controls. This increase is positively correlated with age in healthy controls but not in MS patients and with CD4^+^CD28^−^ T cell numbers in all subjects (63, 67). Remarkably, double negative B cells and ABCs are also increased in the CSF of MS patients (67). Double negative B cells from MS patients have a higher activation potential than those from controls. They are involved in antigen presentation as well as costimulation, and can produce proinflammatory cytokines (TNFa, LTa), and granzyme B after stimulation (67).

#### Tregs

In RR/PPMS, Tregs (CD4^+^CD25^+^CD127^−^) levels were stable as compared to controls, although resting Tregs (CD45RA^+^CD25^dim^) were reduced, while activated Tregs (CD45RA^−^CD25^bright^) were increased in active MS (207, 208). There are some discrepancies whether Treg numbers and expression of surface markers are different between MS and control subjects (207–209). Overall, it is considered that the suppressive activity of Tregs is reduced in (RR)MS, but it seems to improve in SPMS (210–213). Several miRNAs have been found to target the TGFb pathway limiting the differentiation of CD4^+^ naïve T cells to Tregs. These miRNAs, however, did not affect their suppressive function (214).

#### Inflammatory Mediators/Inflammaging

CSF levels of TNFa, CXCL10 and IL8 increased with age in healthy controls, while IL10 level was the lowest in the middle age group (40-to-59-years-old). This inflection point of IL10 production possibly occurs 10–20 years earlier in MS, due to premature immunosenescence, which might correspond to disease onset. On the contrary, in MS, TNFa, CXCL10, IL8, and IL10 levels were higher than in controls, but only IL8 and CXCL10 increased with age. Moreover, there is a shift from Th1 to non-Th1 cytokine profiles in aging and MS, as the age-related increase of CXCL10 was relatively lower than for the other cytokines (41).

Circulating and CSF levels of a few markers are overall in favor of the presence of inflammaging in MS, although there are some discrepancies between studies. IL6 and TNFa are increased in the serum and the CSF in RRMS, mainly relapse, and SP/PPMS. IL6 correlates with disease duration, serum TNFa in PPMS correlates with disease progression. Serum IL10 levels were increased with remission, while CSF levels were high during relapse [reviewed by (215)].

#### Proteostasis/Autophagy

Autophagy is increased in active RRMS, evidenced by the upregulation of ATG5 in peripheral T cells and in encephalitogenic T cells on brain autopsy samples (216). It exhibits both detrimental and protective effects dependent on the cell type. It enhances neuroinflammation by supporting autoantigen presentation by DCs and the survival of autoreactive B and T cells. Conversely it protects neuronal integrity, oligodendrocyte survival and the fragile pro/anti-inflammatory balance in astrocytes and microglia [reviewed by (217)]. However, sustained autophagy due to unresolved damage might lead to its detrimental dysregulation, paradoxical inflammasome activation and apoptosis (162, 218).

#### Telomeres/Telomerase

Telomeres in whole blood DNA (thus mainly PBMCs) were shorter in all MS subtypes (219) as compared to controls, and their length was negatively correlated with age. Telomere shortening was associated with a higher relapse rate, disability, and brain atrophy (220). It was predictive of transition to SPMS (220, 221).

#### Oxidative Stress/Mitochondrial Dysfunction

Peripheral lymphocytes of MS patients exhibit an increased glucose demand with impaired oxidative phosphorylation, alongside mitochondrial dysfunction (marked by a reduced enzymatic activity and a decoupling of the respiratory chain) (222–224). Concurrently, oxidative stress can promote T cell activation and Th17 differentiation (225–227). Interestingly lymphocytic resistance to apoptosis might partly be due to an impaired mitochondria-mediated apoptotic deletion, as observed in CD4^+^CCR5^+^ T cells of PPMS (228).

#### Epigenetics

Contrary to aging, methylation appears to be globally increased in MS [reviewed by (229)], with different methylation profiles between MS phenotypes, higher in PPMS compared to RRMS (230), but slightly higher in RRMS compared to SPMS (231). Lymphocyte signaling, T cell activation and migration were common pathways to RRMS and SPMS methylation profiles, while myeloid cell function and neuronal and neurodegenerative genes and pathways were SPMS-specific (231). Thirteen N6-methyladenosine (m6) regulatory genes were overexpressed in the CSF of MS patients as compared to healthy controls, of which 9 were negatively correlated with age. Remarkably, non-supervision consensus clustering separated RRMS and progressive MS patients in 2 distinct clusters, with higher levels of the m6 regulatory genes and m6 RNA methylation in RRMS patients (232).

miRNAs are upregulated in the CSF of mainly relapsing MS patients and associated to inflammatory (NF-kB, FOXO, TNFa, TGFb), cell cycle and p53 signaling pathways (233). miR-155-5p targets *SOCS1* and hence promotes Th17 and Treg differentiation, and microglia-mediated immune response through expression of IL6, TNFa and induced nitric oxide synthase (iNOS) (234, 235). It also disrupts the BBB while miR-146a-5p protects it by modulating leukocyte adhesion to endothelial cells (236, 237). Moreover, miR-146a-5p inhibits Th17 differentiation by repressing TNF receptor-associated factor *(TRAF)6* and *IRAK1*, transducers of NF-kB (238). Remarkably, miR-150 that targets *CMYB*, promotes terminally effector rather than precursor memory CD8^+^ T cells and is also expressed in mature B cells (239, 240).

### Relevance of Immunosenescence on MS Disease Features

#### Resident Cells of the Central Nervous System

MS-related inflammatory processes influenced by immunosenescence, potentially alter the function of CNS-resident cells by promoting senescence and a pro-inflammatory phenotype, which enhances the oxidative burden, resulting in alteration of mitochondrial function and DNA integrity. Moreover, cell cycle arrest and phenotypic changes in senescent cells might affect their functions and their regenerative capacity (241).

##### Oligodendrocytes

The adult brain encloses its remyelination potential into a pool of oligodendrocyte progenitor cells (OPCs). OPCs represent 5–10% of all CNS cells, can undergo asymmetric division and migrate to the site of demyelination to differentiate into mature oligodendrocytes thereby forming new myelin sheaths. This remyelination potential naturally decreases and slows down with age [reviewed by (241, 242)]. In addition, OPCs are improperly recruited to chronically demyelinated MS lesions and fail to differentiate with disease progression, due to intrinsic and extrinsic factors (243, 244).

Intrinsic factors include age-related decline of histone deacetylation and methylation in OPCs and oligodendrocytes (by reduced HDAC class I expression), enhancing the heterochronic expression of transcriptional inhibitors [e.g., inhibitor of DNA-binding (Id)4] as well as global hypomethylation by downregulation of *Dnmt1* in OPCs of aged mice (245–247). Likewise, DNA methylation of ID2/ID4 allows OPC differentiation, but their methylation levels were lower in MS lesions on human brain samples than in controls (248). Extrinsic factors from the OPC environment can also affect their differentiation. Unlike induced pluripotent stem cell (iPS)-derived neural progenitor cells (NPCs) from age-matched healthy controls, NPCs from PPMS patients expressed senescence markers (p16^INK4^, p53, increased senescence-associated beta-galactosidase activity), and failed to induce OPC differentiation. This was reversed by treating the NPCs (and not the OPCs) with rapamycin or a blocking antibody against high-mobility group box (HMGB)1, a mediator of neuroinflammation in the SASP of NPCs (249, 250).

Moreover, immune reactive OPCs can contribute to neuroinflammation and to their own functional impairment in demyelinating conditions, as they express IL1b, MMP9, MHC-I/II, and immunoproteasome genes, facilitating the early disruption of the BBB, the recruitment of activated immune and glial cells and their production of cytokines (e.g., IL6 by astrocytes) (242, 251–254). They are also involved in neuronal cytotoxicity, by enhancing glutamatergic transmission through IL1b or dysregulation of α-amino-3-hydroxy-5-methyl-4-isoxazolepropionic acid (AMPA) receptors through IFNg, directly or indirectly by inducing lymphocytic cytokines (255–257). Inversely, the SASP of the surrounding cells can interfere with OPC differentiation (258).

##### Microglia

Microglia, maintained in a quiescent state by TGFb (259) and inhibitory ligand-receptor interactions with neurons, astrocytes, and oligodendrocytes, scan their environment through their ramifications, for danger signal and can sense extracellular ATP/UDP changes mirroring neuronal or astroglial injury/activity. Activated microglia will transiently change into pro-inflammatory subsets, particularly during myelin clearance, which sustains inflammation and hinders remyelination, while regulatory subsets support neuroprotection. However, their physiological age-related functional changes decrease their reparative ability toward CNS damage [reviewed by (260)].

Since microglia have a relatively long lifespan and a slow turnover rate, they are more prone to accumulate DNA damage and experience changes during aging [reviewed by (261)]. The motility and ramifications of microglia are reduced, and their sensome gene expression profile changes with age, delaying their recruitment to site and reducing their ability to sense their surroundings. Moreover, aged microglia are chronically activated and exhibit an elevated immunoreactivity and an exaggerated pro-inflammatory response, the so-called microglial priming. TLRs and advanced glycan-end products are upregulated while immune-suppressive factors (CD200R, CX3CR1) are downregulated, enhancing the expression of MHC-II, pro-inflammatory cytokines (IL1, IL6, TNFa), and the production of ROS/reactive nitrogen species (RNS) (by overexpressing NOS and NADPH oxidase) (262–265). Conversely, the age-related increase in TGFb levels, with senescence promoting roles [reviewed by (266)], induces changes in aging microglia that interfere with their ability to acquire a regulatory phenotype and to promote OPC differentiation (267). Moreover, activated microglia initiate a TNFa-mediated synaptic degeneration, and reciprocally influence astrocytes through TNFa and ATP to prompt the astroglial release of glutamate (257, 268).

With age, the phagocytic activity of microglia declines, impairing the clearance of myelin debris and delaying remyelination [reviewed by (269)]. Furthermore, as lysosomal degradation and cholesterol efflux are defective, lipofuscin granules (insoluble aggregates of myelin) accumulate, which increase inflammasome signaling and protein expression (270, 271).

Although microglia and macrophages are phenotypically related and complement one another in MS pathogenesis, their age-related changes partially differ. Aging macrophages are deficient in phagocytosis and chemotaxis, as microglia. Contrarily to microglia, they lose their pro-inflammatory and regulatory functionality (i.e., reduced activation of NF-kB, downregulation of TLR4, TNFa, IL6) [reviewed by (269)]. Interestingly, transferring young macrophages into an aging demyelinating brain enhanced remyelination (272).

##### Astrocytes

Astrocytes are part of the CNS innate immune system and participate to demyelination by impairing the BBB, by controlling the passage of immune cells through the BBB (cellular adhesion molecules), by attracting peripheral immune cells and resident CNS cells to the lesion site (chemokines), by guiding T cell phenotypes, by inducing B cells (BAFF), by modulating microglial recruitment and function, and by acting as APCs. Although astrocytes can prevent excitotoxicity by glutamate uptake, they can worsen it by secreting several cytotoxic factors (ROS, RNS, glutamate, ATP) in response to IFNg and IL1b stimulation. Furthermore, TNFa downregulates glutamate receptors in astrocytes, thus elevating the extracellular levels of glutamate, which is directly toxic to oligodendrocytes, axons and neurons. Astrocytes further secrete fibroblast growth factor 2 (FGF2) and produce glycosaminoglycan hyaluronan, which promote OPC proliferation instead of their differentiation, and produce chondroitin sulfate proteoglycans, ephrins, and myelin-associated inhibitors, which inhibit axonal growth. The glial scars formed by reactive astrogliosis try to contain the demyelination by surrounding the damaged area, but their rigidity hinders remyelination and axonal regeneration [reviewed by (273)].

In aging astrocytes, the overexpression of the intermediate filaments, glial fibrillary acidic protein (GFAP) and vimentin, parallels increased p16^INK4^ expression and cell cycle arrest. Moreover, TGFb1 and HMGB1 induce pro-inflammatory cytokines (IL6, TNFa, IL1b, prostaglandins) and chemokines constituting the SASP of aging astrocytes (274, 275). Interestingly, EAE improves by blocking HMGB1 in the CNS (276). Furthermore, during EAE, oxidative stress sustains excitotoxicity (273, 277).

#### Inflammatory Activity vs. Progression in Relationship to Aging

While 80–85% of patients present with RRMS at a younger age, the relapse rate is reduced with aging. Moreover, the post-relapse recovery potential decreases with age. The decline in white matter integrity and neuro-axonal reserve might precipitate the onset of progression, and increase the risk of accumulating disability (278–281). It is now established that subclinical neurodegeneration starts long before clinical progression becomes more evident, explaining the occurrence of progression independent of relapse activity (PIRA) in earlier phases of the disease (282). Therefore, according to natural history studies, up to 50% of RRMS patients might transition to SPMS, 15–20% present with disability progression from onset (PPMS) (18). Remarkably, both PPMS and SPMS onset occurs on average around the age of 45 years. Transition to SPMS happens independently of the duration of the prior relapsing course (283). Aging and underlying senescence might therefore, at least partially, be involved in the evolution and pathogenesis of the disease.

The CNS inflammatory infiltration and acute axonal injury are negatively correlated with age, while in inactive progressive MS, the CNS inflammation declines to the same level as in healthy controls (284). While RRMS is characterized by a disrupted BBB allowing the invasion of the CNS by peripheral immune cells, progressive MS is characterized by a compartmentalized CNS inflammation, behind a closed BBB [reviewed by (285, 286)]. Follicle-like structures, enriched in B and plasma cells, form in the meninges and these cells have a higher relative contribution within the infiltrates (287). Perivascular and parenchymal T/B cell infiltrates are limited. New active lesions are infrequent. Slowly expanding white matter lesions, also called smoldering lesions, with low-grade myelin destruction and axonal degeneration, are formed by a moderate lymphocytic infiltration and a dense network of reactive astrogliosis in their center (288, 289), surrounded by activated microglia and macrophages forming a narrow rim (290). Cortical lesions are frequent and are also mainly caused by activated microglia, resulting in synaptic and neuronal loss (285, 286). In the normal appearing white matter, the proinflammatory state induces microglial and astrocytic activation resulting in diffuse axonal injury (291).

During the progressive phase of the disease, the oxidative burst by activated microglia is prominent (285). Iron accumulates with age in the brain and is stored with ferritin in oligodendrocytes. The oligodendrocytes, harmed by inflammation/oxidation, release ferrous iron. Ferrous iron (Fe^2+^) reacts with H_2_O_2_ to form ferric iron (Fe^3+^) and a hydroxyl radical, which increases the oxidative stress (285). Ferric iron is incorporated by microglia and macrophages at the active lesion margins, forming the magnetic rim lesions detectable by MRI in about 50% of the cases (290). This iron uptake causes dystrophy of macrophages and microglia, leading to the secondary release of iron and fueling the oxidative stress. Although autophagy is increased in progressive MS in an attempt to ensure cellular homeostasis, it is not enough to compensate the mechanisms at play in the periplaque environment causing cellular senescence (216, 285). Moreover, the oxidative stress results in and is subsequently amplified by mtDNA damage and mitochondrial dysfunction of the respiratory chain complexes. Furthermore, synaptopathy, which happens also in normal aging brain, is caused by reduced neurotrophic factors and excitotoxicity resulting from a glutamatergic/gamma-aminobutyric acid (GABA)-ergic imbalance, as well as by pro-inflammatory cytokines (IL1b, IL6, TNF) of activated microglia, astroglia, and infiltrating lymphocytes (257, 292, 293). IL1b can also alter synaptic plasticity. Both are exacerbated by neuroinflammation and accelerated with age during MS [reviewed by (293)]. These features contribute to neurodegeneration and translate at the macroscopic level into accelerated brain atrophy, which can be viewed as premature aging of the MS brain, at a rate of 0.7–1% per year, compared to 0.1–0.3% per year in healthy subjects (285, 294, 295).

Exosome-associated miR-15b-5p/23a-3p/30b-5p/223-3p/342-3p/374a-5pand miR-432-5p/433-3p/485-5p are, respectively, up- and downregulated in RRMS vs. PP/SPMS (296). Interestingly, miR-15b-5p and -23a-3p are predicted to target *FGF2*, a promoter of OPC migration present in active and in the periphery of chronic lesions and elevated in the CSF of RR/SPMS (297–299). miR-342-3p is required for NF-kB induction in TNFa-activated microglia (300). miRNAs dysregulated in cortical lesions as compared to myelinated gray matter, are involved in axonal guidance, TGFb, and FOXO signaling. Furthermore, miR-20a/25/29c/149^*^ are associated to gray matter atrophy (301).

**Figure 2:**
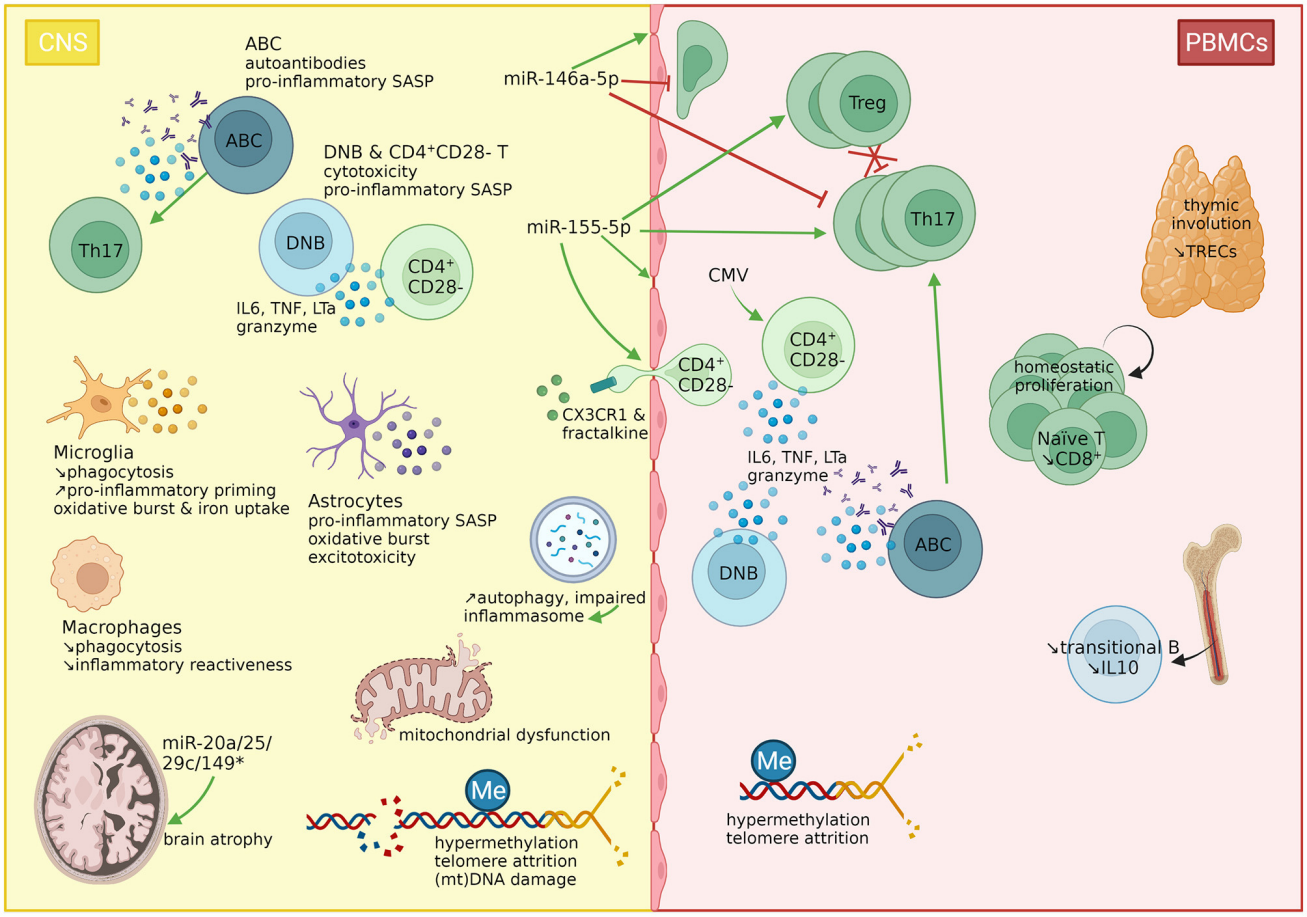
immunosenescence features in multiple sclerosis. Features of immunosenescence have been described in MS. The thymic involution (measurable by reduced TREC levels) induces the homeostatic proliferation of T cells. However, naïve T cells rapidly differentiate into memory T cells due to antigenic stimulation. While Th17 cells, and to a lesser extent, Tregs are expanded in the periphery, the latter fail to suppress Th17 cells. A subset of CD4^+^ T cells has lost the costimulatory signal CD28, marking the T cell exhaustion mainly through sustained CMV stimulation. These cells express the CX3CR1 receptor, favoring their migration through the BBB, as their ligand, fractalkine, has been found overexpressed in the cerebrospinal fluid (CSF). The B cell compartment is characterized by a reduction in immunoregulatory transitional B cells, but an increase in double negative B cells (DNBs) and ABCs. These three subsets (CD4^+^CD28^−^ T cells, DNBs, ABCs) have been linked to immunosenescence and detected in the CNS of MS patients. CD4^+^CD28^−^ T cells and double negative B cells produce TNFa, and granzyme B. CD4^+^CD28^−^ T cells produce also IL6, and double negative B cells produce LTa, hence corresponding to the senescence-associated secretory phenotype of these cells. ABCs produce TNFa and autoantibodies and polarize Th17 cells. In the CNS, both microglia and macrophages have impaired phagocytic properties, but while microglia are primed, macrophages lose their inflammatory reactiveness. Microglia further produce ROS and incorporate iron. Astrocytes produce also proinflammatory cytokines and ROS and release glutamate, inducing excitotoxicity. The oxidative burst causes mitochondrial dysfunction, and (mitochondrial) DNA damage. Autophagy is increased but impaired, which might induce the inflammasome. Moreover, telomeres shorten with age and disease progression. Interestingly, hypermethylation is a common feature in PBMCs from MS patients, found in several subsets as well as in brain tissues, while hypomethylation has on the contrary been linked to aging. Finally, miR-146a-5p and miR-155-5p are two major immuno-microRNAs with opposite effects on the integrity of the BBB, T cell migration and the differentiation of Th17 cells. Herein, miR-155-5p displays pro-inflammatory characteristics, but also supports the differentiation of Tregs. Four miRNAs (miR-20a/25/29c/149*) have been linked to brain atrophy. CMV, cytomegalovirus; CNS, central nervous system; CX3CR1, C-X3-C Motif Chemokine Receptor 1; B, B cell; ABC, age-associated B cell; DNB, double negative B cell; IL, interleukin; LTa, lymphotoxin A; Me, methylation; mt, mitochondrial; MS, multiple sclerosis; PBMCs, peripheral blood mononuclear cells; SASP, senescence-associated secretory phenotype; T, T cell; Th, T helper cell; TRECs, T cell receptor excision circles; Treg, regulatory T cell; TNF, tumor necrosis factor. Created with BioRender.com.

In summary (Table 1, Figures 1B, 2), with disease progression, the involvement of the peripheral immune system becomes secondary, while increasing oxidative stress, sustained by the pro-inflammatory phenotype of glial cells, is the major mechanism causing mitochondrial dysfunction in all CNS-resident cells, inducing their complete functional decline (impaired clearance of myelin debris, impaired remyelination, energy failure, loss of neurotrophic support, release of neurotoxic factors), resulting in irreversible neurodegeneration.

**Table 1:** Comparison between immunosenescence features in aging, SLE, RA, and MS.

	**Immunosenescence features**	**Physiological aging**	**MS**	**SLE**	**RA**
Innate immunity	NK cells	↘ CD56^bright^ ↗ CD56^dim^ Impaired functions	↗ CD56^bright^ in CSF (RRMS) ↗ CD56^dim^ in serum (PP/SPMS) Impaired functions	↘ absolute numbers Impaired immunoregulatory function	↘ numbers Impaired immunoregulatory function
	Neutrophils	↘ NETosis/phagocytosis	↗ NETosis, ↘ apoptosis ↗ in CSF (onset/early in relapse)	↗ NETosis, neutropenia	↗ NETosis
	Monocytes	↘ classical, ↗ non-classical	↗	↘ non-classical	↗ non-classical, ↗ CD56^+^
	Macrophages	↘ phagocytosis/APC function ↘ proinflammatory	Macrophages: ↘ proinflammatory (PMS) Microglia: ↗ proinflammatory	↗ proinflammatory	↗ proinflammatory
	Phagocytosis	↘	↘ (microglia & macrophages, PMS)	↘	↘
Adaptive immunity	Thymic output	↘	↘	↘	↘
	TCR repertoire	↘	↗	↘	↘
	T helper	↘ Th1, ↗ Th2 cytokines ↗ Th17	↗ Th1 ↗ Th17 ↘ Th1, ↗ Th2 cytokines with age	↘ Th1 cytokines ↗ Th17 ↗ IL17-producing DNT	↗ Th1 ↗ Th17 (in early stages)
	Memory T cells	↗	↗	↗	Unchanged T_EM_ in blood ↗ T_EM_ (CD8+) in SF
	Terminally differentiated CD4^+^CD28^−^	↗ related to CMV infection	↗	↗	↗
	CD4^+^/CD8^+^ ratio	<1	>1, especially in CSF	<1	>1 in blood <1 in SF
	Treg numbers suppressive function	↗ ≈ (vs. Th1), ↘ (vs. Th17)	≈/↗ ↘	↘ ↘	↘ in blood (active RA), ↗ in SF ↘
	Immature B cells	↘ transitional B cells, impaired function	↘ transitional B cells, impaired function Present in CSF	↗ transitional B cells	↘ transitional B cells impaired function (active RA)
	Memory B cells class-switched IgD^−^CD27^+^/unswitched IgD^+^CD27^+^	Unchanged	Unchanged in blood ↗ class-switched in CSF (adult MS) ↗ unswitched in CSF (pediatric MS)	↗ class-switched ↘ unswitched	↘ class-switched
	Double negative B cells	↗	↗ in <60 years-old, in blood/CSF	↗	↗
	ABCs	↗	↗ in <60 years-old, in blood/CSF	↗	Detected in SF
SASP	Inflammaging	IL6/8, CRP, TNFa	↗ IL6 and TNFa in serum/CSF (relapse, SP/PPMS) ↗ IL10 in serum (remission), in CSF (relapse)	↗ TNF, IL6, IL18, IFN-I ↗ IL10, IL15, BAFF	↗ IL6, CRP, TNFa (serum) ↗ TNFa, MMP1, MMP3, ↘ IL10 (SF)
Other senescence features	Oxidative stress	↗	↗↗	↗↗	↗↗
	Autophagy	↘	↗ but impaired	↗ but impaired	↗ but impaired
	Telomere length	↘	↘	↘ Except in CD8^+^CD28^−^	↘
	DNA methylation	↘	↗	↘	↘

*APC, antigen presenting cells; BAFF, B cell activating factor; CMV, cytomegalovirus; CRP, C-reactive protein; CSF, cerebrospinal fluid; DC, dendritic cells; DNT, double negative T cells; IFN, interferon; IFN-I, type I interferon; IL, interleukin; MMP, matrix metalloproteinase; MS, multiple sclerosis; NET, neutrophil extracellular traps; NK, natural killer cells; PP/SPMS, primary/secondary progressive MS; RA, rheumatoid arthritis; RRMS, relapsing-remitting MS; SLE, systemic lupus erythematosus; SASP, senescence-associated secretory phenotype; SF, synovial fluid; TCR, T cell receptor; T_EM_, effector memory T cell; Th, T helper; TNF, tumor necrosis factor; Treg, regulatory T cells. ↗, increased; ↘, decreased; and ≈, approximately equal*.

#### Efficacy and Safety of Disease Modifying Therapies in Aging MS Patients

Disease modifying therapies (DMTs) are efficient to reduce clinical relapses and radiological disease activity in active MS. However, due to the predominant CNS-restricted inflammation concurrent to neurodegeneration, treatments for progressive MS remain scarce, possibly more effective to patients with superimposed active inflammation [reviewed by (302)]. Moreover, since clinical trials classically exclude patients over the age of 55 years, the safety and efficacy of DMTs in older MS patients is still debated, while these patients represent a growing proportion of the MS population (17, 302). In patients younger than 40.5 years, high-efficacy drugs (ocrelizumab, mitoxantrone, alemtuzumab, and natalizumab) initiated without delay, were more powerful than lower-efficacy drugs (fingolimod, dimethyl fumarate, interferon-beta, teriflunomide, and glatiramer acetate), but may already lose their benefits on disability progression after that age. However, this model could not distinguish benign from active MS courses. The same meta-analysis by Weideman et al. found DMT efficacy to be negatively correlated with age and predicted no efficacy of DMTs after the age of 53 years (303). Moreover, the intrinsic effects of DMTs on immune cells in addition to the age-related changes in the immune system might become deleterious for remyelination and immunosurveillance with age, since DMTs deplete, sequestrate or functionally impair lymphocytes (191, 304–307). Discontinuing DMTs in the elderly might be reasonable for these reasons, however, studies are sparse. Herein, stable patients discontinuing DMTs experienced the same time to relapse as patients still on DMTs but a shorter time to disability progression. The latter was also correlated with age. However, this study included MS patients from 18 years and older, and did not focus specifically on the elderly MS patients (308). For patients older than 45 years, a 4-year relapse-free disease course under DMTs was predictive of absence of relapse following DMT discontinuation while longer disease duration and higher EDSS were predictive of disability progression (309).

Since DMTs directly act on the immune system, there has always been a major concern about risk for cancer and infections (191, 310). While some found initially a slightly increased risk for cancer (e.g., urogenital, breast, CNS cancers, lymphomas, melanomas) (311, 312), the overall risk considering all current DMTs is not increased [reviewed by (313)], although a higher incidence of neoplasm with depletive DMTs (alemtuzumab, cladribine, ocrelizumab) was found by a meta-regression analysis, especially after the age of 45 years (314). Switching from DMTs, especially more than twice, was also a risk factor for cancer (311). Furthermore, awareness is raised for the possible link between natalizumab, fingolimod, cladribine, or alemtuzumab and several types of immune malignancies, melanomas, carcinomas *etc*. [reviewed by (313)].

Natalizumab, dimethyl fumarate, and fingolimod increase the risk of progressive multifocal leukoencephalopathy (PML) caused by JC-polyomavirus. The risk related to natalizumab remains for several months after switching to another DMT, which explains carry-over cases as seen with ocrelizumab, fingolimod, or teriflunomide. Age, later age at DMT initiation (>50 years), prior immunosuppressive treatment, and lymphopenia, particularly inside the CNS, are important risk factors for PML, although cases with normal blood lymphocyte count have been reported (310, 315–317).

Other opportunistic infections increasing with age can be cryptococcal meningitis and herpes encephalitis (fingolimod, natalizumab) (318, 319), mucocutaneous herpes infection [sphingosine-1-phosphate receptor modulators, natalizumab, alemtuzumab], and varicella-zoster reactivation [sphingosine-1-phosphate receptor modulators, dimethyl fumarate (320), natalizumab, cladribine, alemtuzumab, ocrelizumab], human papilloma virus (fingolimod), and Listeria meningitis (alemtuzumab) [reviewed by (310, 321)].

Most DMTs act through different mechanisms on the subsets of the adaptive immune system that might undergo immunosenescence in parallel. For example, DMTs have been found to differentially affect the thymic and bone marrow output after treatment initiation but also in an age-related fashion. Both are, respectively, measured through TRECs, which decrease with age in healthy control, and K-deleting recombination excision circles (KRECs), which remain stable. While TREC levels did not change between DMT-naïve and DMT-discontinued patients, KREC levels were significantly enhanced in the latter. Interestingly, fingolimod that sequestrates lymphocytes in lymph nodes, showed a reduction in thymic and bone marrow output at 6 and 12 months after treatment initiation while the opposite was observed in natalizumab, that sequestrates lymphocytes in blood vessels, apart from the inflammation site. With the immunomodulatory IFNb both thymic and bone marrow output were stable within the first months, while KREC levels did not further decrease with age, contrary to what was observed in patients with fingolimod or natalizumab. With alemtuzumab, which temporarily depletes peripheral lymphocytes to induce their repopulation, only KREC levels increased following treatment and both parameters further remained stable with age (322).

In summary, the MS population is growing older, concurrently accumulating the comorbidities related to aging. The efficacy and safety of DMTs seem to decrease with age, although robust data are still missing. DMTs might increase the risk of opportunistic infections and cancers in the elderly by changing immune cell population distributions and by affecting the functions of the immune system, hence possibly promoting certain immunosenescence features, in combination with MS-related premature immunosenescence.

### Immunosenescence in Systemic Lupus Erythematosus

SLE pathogenesis is characterized by Th17 polarization, autoreactive B cells producing autoantibodies targeting nucleic acid-bound antigens and the innate immune system providing a strong IFN-I signature (323). The tissue damage in SLE, resulting in organ dysfunction (e.g., kidney, brain, lungs, cardiovascular system) corresponds to premature biological aging. The immune dysregulation in SLE presents some features resembling immunosenescence, mainly in the adaptive immune system, but underlying mechanisms might be different. In the innate immune system, the effects of SLE and aging appear to be more divergent [reviewed by (20)].

#### Innate Immunity

Unlike immunosenescence, SLE-derived neutrophils primed by IFNa and autoantibodies produce more ROS and are engaged in NETosis, which causes tissue damage and partly explains the neutropenia observed in this disease (324–326). Moreover, the non-classical monocytes (CD14^+^CD16^++^), present in a decreased proportion in SLE contrarily to aging, display a reduced phagocytosis capacity, but an increased expression of TLR, TNFa and IL10 (20, 327). Macrophages contribute to SLE by their defective phagocytosis of apoptotic cells, their polarization toward a proinflammatory phenotype, and an aberrant activation of their autophagy and inflammasome machinery (328). The imbalance between the decreased immunoregulatory (CD56^bright^) and the increased pro-inflammatory function (CD56^dim^) of NK cells is correlated with disease activity, although their relative frequencies are unchanged, while their absolute numbers are decreased (329, 330). Moreover, increased serum levels of IFNa in active SLE parallel the frequency of IFNg-producing NK cells (330) [as seen in a TNFa/IL12-mediated viral infection response (331, 332)].

#### T Cells

Thymic output and TCR repertoire are reduced in SLE (333, 334). SLE patients often exhibit the key features of the immune risk phenotype, an inverted CD4^+^/CD8^+^ ratio, due to CD8^+^ T cell expansion and a higher CD4^+^ T cell turnover (335). Th17 and IL17-producing double negative T cells are involved in SLE pathogenesis [reviewed by (336)]. A dominant granzyme-producing CD8^+^ T cell population is found in patients with severe nephritis, leucopenia, and clinically active disease (337, 338). Expanding CD4^+^CD28^−^ T cells produce IFNg in moderately active SLE and are positively correlated with the clinical disease score (339). Conversely, some autoreactive CD8^+^CD28^−^ clones secrete less IFNg and comparatively, relatively more IL10 but with impaired suppressive capacities (340). Remarkably, TCR signaling is driven by the FCgR chain in SLE rather than the TCR-zeta chain, due to its altered composition, resulting in a lower activation threshold, higher calcium influx, increased excitability and baseline stimulation [reviewed by (341)].

#### B Cells

Contrary to aging, there is a shift toward immature B cells in SLE, due to a two-fold increase in transitional B cells with a defective tolerance checkpoint resulting in autoreactive B cells producing autoantibodies (342, 343). Frequent cycles of B cell activation and differentiation shape peripheral B cells, marked by an expansion in switched (IgD^−^CD27^+^) memory B cells and double negative (IgD^−^CD27^−^) B cells, as well as a subset of activated memory B cells (IgD^−^CD27^−^CD95^+^CD21^−^), the latter being increased during disease flares (344–347). The CD11^high^TBET^+^ ABCs as well as two ABC-like subtypes, found in African American patients, have been linked to disease severity (348, 349). Double negative type 2 CXCR5^−^ cells with a unique cytokine, cytokine receptor, transcription factor and signaling factor expression profile, are increased in young patients, and do not further expand with age (350).

#### Tregs

Data on the number and function of Tregs in SLE are very disparate, mainly due to study population and staining strategy differences. However, Tregs appear to be largely outpaced by the T and B cell activation in SLE, possibly due to the decrease in IL2 production, that mediates Treg homeostasis, and the increase in IL6 production, that induces effector T cell activation. Furthermore, the Th17/Treg ratio increases in SLE alongside the decrease of TGFb. Finally, the effect of IL10 remains unclear as it has both anti-inflammatory effects (when produced by Tregs and type 1 T-regulatory cells) and it induces autoreactive B cell proliferation (when produced by monocytes and B cells) [reviewed by (351–353)].

#### Inflammatory Mediators Related to Inflammaging

The binding of immune complexes induces the production of pro-inflammatory inflammaging-associated cytokines, such as TNF, IL6, and IL18 by monocytes/macrophages, but also of IFN-I by plasmacytoid DCs, and immunoregulatory cytokines IL10, IL1, and BAFF. Interestingly, they have been linked to disease activity, while CRP for instance is not [reviewed by (354)].

#### Proteostasis/Autophagy

Contrary to aging, autophagy is increased in SLE, as highlighted by the increased autophagy-associated markers in naïve CD4^+^ T cells (355). Moreover, the autophagosome density of B cells is positively correlated with disease activity (356). However, while autoantibodies from lupus patients can induce autophagy in T cells of healthy controls *in vitro*, T cells from SLE patients are resistant to it (355).

#### Telomeres/Telomerase

Overall, telomere length is shorter compared to aged-matched healthy controls in SLE patients, with the reduction being even more pronounced in younger subjects, and without the typically progressive age-related decline seen during physiological aging. On the contrary, telomerase activity is increased in T and B cells. However, this fails to compensate for the accelerated telomere attrition in T cells (357, 358). Telomerase activity, but not telomere length, is positively correlated with disease activity (357). Unlike what is observed during aging, CD8^+^CD28^−^ T cells in SLE have longer telomeres, an increased telomerase activity and a preserved proliferative potential (359).

#### Oxidative Stress/Mitochondrial Dysfunction

Activated SLE T cells produce an excess of ROS and RNS and deplete the glutathione reserve, which leads to mitochondrial dysfunction (166, 360, 361). Oxidated DNA and mtDNA damage are correlated with the high serum levels of cytokines (IL10, IL23, IFNa, IFNg) and chemokines (CXCL10 and MCP1) (362). Remarkably, only higher mtDNA damage levels are related to disease duration (363, 364). Moreover, peroxynitrite-modified histones, due to amino-acid nitration by RNS, induce high titers of anti-histone antibodies and UV-induced DNA damage potentially induces IFN-I (365, 366).

#### Epigenetics

DNA methylation levels are globally decreased in T cells from SLE patients. Herein, the hypomethylation of interferon signature genes [*i.a*. interferon regulatory factor (IRF)5, IRF7] is a hallmark of SLE pathogenesis and immune response genes are associated with chromatin remodeling (e.g., trimethylation of H3K4) (367–369). Moreover, nitration of the protein kinase C (PKC/ERK pathway) inhibits its delta catalytic activity, resulting in decreased activity of DNMT1 and thus low methylation levels in CD4^+^ T cells allowing the transcription of *CD70*, possibly *CD11a*, and perforin (370–372). Interestingly, miR-21/29b/126/148, which are overexpressed in CD4^+^ T cells from SLE patients, downregulate *DNMT1* (373–375), while miR-199a-5p increased splenic CD4^+^ T cell senescence by inhibiting *SIRT1* and thus increasing the acetylation and consequently the activation of p53 (376). Upregulation of miR-7 and -30a in B cells of SLE ensures B cell proliferation, differentiation to plasma cells and antibody production (377, 378), while miR-15a activates the apoptosis of Bregs by targeting *BCL2* (379, 380). Furthermore, miR-142 downregulation by histone and DNA methylation of its regulatory region, results in the activation of T cells, hyperstimulation of B cells and suppression of Treg function (381, 382). miR-146a inhibits and miR-155 enhances IFN-I (383, 384). Finally, miR-125a stabilizes Treg-mediated homeostasis but is downregulated in SLE, and mir-31 and -34a, induced by the NF-kB pathway, target *FOXP3* (385–388).

### Immunosenescence in Rheumatoid Arthritis

RA is likely due to a systemic immune dysregulation, possibly driven by DCs and macrophages that present citrullinated antigens to autoreactive T cells, inducing the production of pro-inflammatory and joint damaging mediators and causing synovial inflammation, and articular and extra-articular tissue damage (389, 390). A premature senescent phenotype of immune cells has been evidenced in RA and partly linked to its pathogenesis [reviewed by (21)].

#### Innate Immunity

Neutrophils are involved in generating citrullinated autoantigens that are afterwards externalized by NETosis, while anti-citrullinated protein antibodies promote NETosis. Neutrophils in joints also produce cytokines and ROS and release proteases by degranulation (391). Intermediate (non-classical) pro-inflammatory CD14^+^CD16^+^ monocytes were similarly increased in elderly subjects with atherosclerosis and middle-aged RA patients as compared to young healthy controls (392, 393). Young RA patients have also a higher frequency of CD56^+^ monocytes, producing more TNFa, IL10, IL23, and ROS, although this is normalized by TNFa blocking therapy. Interestingly, in RA patients, the age-dependency of circulating CD56^+^ monocytes is lost (394). Overall, macrophages drive joint inflammation in RA by secreting cytokines, chemokines and tissue degrading enzymes, activating fibroblast-like synoviocytes (FLSs) and promoting T cell infiltration and osteoclastogenesis. However, it is currently unknown how the heterogeneity between infiltrating monocyte-derived and tissue-resident macrophages might impact disease pathogenesis (395). Furthermore, in RA, the NK cells are reduced in number and functionally impaired, seen their increased production of ROS and proinflammatory cytokines, hence hindering their immunoregulatory properties (396, 397).

#### T Cells

The alteration in T cell homeostasis occurs early in RA and is independent of disease duration (398, 399). The proliferative capacity of CD34^+^ HSCs in the bone marrow is reduced due to a decreased ERK signaling pathway. Both CD34^+^ HSCs and naïve CD4^+^ T cells from RA patients are more susceptible to apoptosis, hence reinforcing the burden on homeostatic proliferation in the periphery (398, 400–402). RA patients have a thymic output of healthy individuals aged 20–30 years older. TREC levels are already lower than normal in young RA patients (159, 399). The TCR repertoire is prematurely contracted in both naïve and memory T cells (403). The CD4^+^/CD8^+^ ratio is increased in the blood, and inverted in the synovial fluid (404). Th17 cells are possibly increased in the blood of RA patients and might be more important at early stages of the disease [reviewed by (405)]. Memory T cells are unchanged in the periphery as compared to controls. However, effector memory CD8^+^ T cells are increased in the synovial fluid (404).

Circulating CD4^+^CD28^−^ T cells produce higher levels of TNFa and IFNg than the CD28^+^ T cells. Their rate is correlated with disease severity and the extent of extra-articular manifestations (406, 407). These cells easily react to neoantigens as they express *de novo* NK receptors (CD56, NKG2D) in RA patients (408, 409). Interestingly, i*n vitro* generated CD56^+^CD28^−^ T cells by repeated stimulation of CD56^−^CD28^+^ T cells of young healthy donors, expressed BCL2, p53, and p16^INK4^ that induce cell cycle arrest, a hallmark of cellular senescence, and activated the NF-kB pathway (410). Since they also express CX3CR1, they can migrate into the synovial fluid where FLSs express the ligand fractalkine, induced by TNFa and IFNg. This interaction activates the CD28^−^ T cells, induces the expression of pro-inflammatory cytokines and facilitates the proliferation of FLSs and the secondary activation of T cells (411). The expansion of CD4^+^CD28^−^ T cells in RA patients has been associated with the HLA-DR4 risk factor and a TNFa polymorphism, alongside increased TNFa and IFNg production (159, 412).

#### B Cells

The effect of senescent B cells is not well-described in RA (21). However, transitional B cells are reduced and have impaired functions in PBMCs of active RA (413). Moreover, double negative B cells were increased in the periphery and ABCs have been detected in the synovial fluid of RA patients (414, 415).

#### Tregs

Although discrepancies exist, Tregs appear to be reduced among the PBMCs in active RA, and normalized during remission as compared to controls (416, 417). In the synovial fluid they are overall increased but functionally impaired (417, 418). Interestingly, a novel subset of senescent CD28^−^ Treg-like cells, characterized as CD4^+^FOXP3^+^CD28^−^, was discovered in the blood and synovial fluid of RA patients. They express markers such as CD25, cytotoxic T-lymphocyte-associated protein (CTLA)4 and FOXP3, and also exhibit premature senescence, as shown by reduced TREC levels and an accumulation of phosphorylated gamma-H2AX (upon DNA double-strand break). Moreover, their SASP consists of high levels of pro- (TNFa, IFNg, IL2, IL4, IL17) and anti-inflammatory (IL10) cytokines. However, CD28^−^ Treg-like cells had also impaired suppressive capacities. They could be obtained *in vitro* by stimulating CD28^+^ Tregs with TNFa. Although CD28^−^ Tregs numbers correlated with age, nor CD28^−^ nor CD28^+^ Tregs correlated with disease duration and clinical features (419).

#### Inflammatory Mediators/Inflammaging

Similarly to inflammaging in the elderly, RA patients have increased systemic levels of pro-inflammatory cytokines (IL6, CRP, TNFa) (420). In the synovial fluid TNFa, MMP1, and MMP3 levels were increased in early and established RA, IL10 decreased during established RA only as compared to osteoarthritis (421). Moreover, RA-derived FLSs produce more IL6, IL8, vascular endothelial growth factor, and prostaglandin E2 in response to IL1b during *in vitro* induced senescence (422). Both TNFa and IL6 play a major role in activating effector cells, inducing cytokine/chemokine/autoantibody production and tissue damage [reviewed by (389)].

#### Proteostasis/Autophagy

While autophagy decreases with age, it is increased in RA FLSs due to stress-induced endoplasmic reticulum hyperactivity and an elevated protein turnover, but the ubiquitin-proteasome system is impaired in RA (423, 424). This altered proteostasis may enhance inflammation.

#### Telomeres/Telomerase

The increased telomerase activity of HSCs is insufficient to compensate for telomere shortening (400, 401). Naïve T cells fail to induce telomerase activity following antigen priming. Telomere attrition was observed in granulocytes, naïve and memory T cells (398, 399, 425). Interestingly, telomerase activity of infiltrating cells correlated with synovial lining hyperplasia, but was independent of disease duration or severity (426). The HLA-DR4 risk factor in RA induces premature immunosenescence by accelerating telomere shortening (425).

#### Oxidative Stress/Mitochondrial Dysfunction

Naïve and memory T cells from RA patients have high levels of DNA double-strand breaks due to impaired DNA repair mechanisms [e.g., reduced DNA repair kinase ataxia telangiectasia mutated (ATM)] (427). Moreover, T cells isolated from RA patients enhance the activity of the DNA-dependent protein kinase catalytic subunit (DNA-PKcs), a DNA repair enzyme. The DNA-PKcs-Janus kinase-axis causes chronically cellular stress and intensifies the inflammatory activity of T cells (428). Cell-free mtDNA, released by tissue damage, was found in plasma and synovial fluid of RA patients. Interestingly, intra-articular injection of oxidized mtDNA in mice caused arthritis (429). MtDNA damage in RA (induced by TNFa and ROS) is positively correlated with macroscopic synovitis, and synovial TNFa and IFNg levels, but does not depend on age (430). Furthermore, p16^INK4^ and p16 ^INK4^-encoding genes along with IL6 could be induced in FLSs by H_2_O_2_ or TNFa (431). Likewise, p53 was upregulated in synovial tissues from early and late-stage RA as compared to normal synovial tissue (432). Interestingly p53 mutations, secondary to chronic oxidative stress, have been detected in RA synovial tissue and promoted clonal FLSs expansion and IL6 expression (433).

#### Epigenetics

Epigenetic changes in RA promote the pro-inflammatory profile involved in disease pathogenesis. Global hypomethylation, along with a decrease in active DNMT1 in the FLSs was found in RA (434). Hypomethylation in PBMCs is correlated with the disease activity score (435). The promoter gene of IL6 and TNFa is hypomethylated in PBMCs and in peripheral naïve CD4^+^ T cells, respectively (436, 437). The IFNg locus is hypomethylated in CD4^+^CD28^−^ T cells as compared to CD28^+^ counterparts resulting in increased expression of IFNg and TNF in the periphery and of IL17, CXCR3, CCR6 in the synovial fluid (406). Histones are globally hyperacetylated by decreased HDAC activity in RA, in particular H3 acetylation in the IL6 promoter was increased in the FLSs (438, 439). TNFa-mediated SIRT1 overexpression in FLSs induced IL6 and IL8 expression and protected cells from apoptosis (440). Moreover, miR-16 and -146a are elevated in synovial fluid, plasma and PBMCs of RA patients and are linked to disease activity (441, 442). Interestingly, HDAC downregulation restored the expression of miR-16 (443). miR-146a and -155 were upregulated in FLSs of RA, both induced by TNFa and IL1b. While miR-155 appeared to be compensatory to joint destruction by reducing MMP1/3, the role of miR-146a in FLSs is unknown, but in PBMCs it fails to properly repress *IRAK1/TRAF6* and thus the NF-kB pathway (444–446).

### A Premature Immunosenescence in Autoimmune Diseases? A Comparison Between Physiological Aging, MS, SLE, and RA

We have compared key features of immunosenescence occurring in physiological aging with changes of the immune system evidenced in MS, SLE, and RA (Table 1). While some features of immunosenescence are found in the 3 AIDs, others differ from physiological aging but also between them. Hence, it seems still unclear whether these findings are inherent to the disease course or causative of its pathogenesis.

In MS (Figure 2), innate immunity does not seem affected by senescence, at least in the early stages of the disease. However, the role of NK cells remains debated, and neutrophilic NETosis is increased contrarily to aging. Macrophages that are strongly involved in the neuroinflammatory processes in the early stages of the disease, lose their function with aging, while microglia remain highly primed. T and B cells display some immunosenescence features: CD4^+^CD28^−^ T cells, ABCs, and double negative B cells are expanded, and exhibit properties supporting autoreactivity. However, the inversion of the CD4^+^/CD8^+^ ratio is missing. Memory Tregs are increased, but their functionality is presumably reduced. In SLE, the innate immune function strikingly differs from what is observed during aging (increased NETosis, decreased non-classical monocytes, a proinflammatory macrophage shift). However, T and B cells display immunosenescence features (CD4^+^/CD8^+^ inversion, CD4^+^CD28^−^ T cell, ABC, ABC-like and double negative B cell expansion), contrarily Tregs are possibly reduced in number and function. In RA, innate immunity shows features of immunosenescence in monocytes as well as a reduced immunosurveillance by NK cells. Macrophages have reduced phagocytic properties despite actively contributing to the inflammation as do neutrophils through increased NETosis. The T cell compartment is marked by an expansion of CD4^+^CD28^−^ T cells, including functionally impaired CD28^−^ Tregs, which are possibly involved in RA pathogenesis. The CD4^+^/CD8^+^ ratio is inversed in the synovial fluid but not in the blood. Double negative B cells and ABCs have been detected in RA, but are barely characterized.

The released inflammatory mediators (e.g., IL6, IL10, TNFa) contribute to the disease pathogenesis of all 3 AIDs and even mirror disease activity in MS. In all 3 AIDS, telomeres are shortened, except in CD4^+^CD28^−^ T cells in SLE, which consequently have a preserved proliferative potential. Mitochondrial dysfunction is increased as is observed in physiological aging, and dysregulated miRNAs are largely involved in inflammatory pathways. However, contrary to aging, autophagy is increased; but impaired. Distinctively, MS appears to feature a global hypermethylation, with distinct clusters between the disease subtypes, rather than the hypomethylation observed during physiological aging as well as in SLE and RA.

## Conclusion

Immunosenescence encompasses functional and phenotypic changes within the immune system occurring naturally during aging. Among other features the resulting loss of self-tolerance, alongside inflammaging might be involved in the pathogenesis of AIDs. In this review, we have discussed and compared the similarities and discrepancies between hallmarks of immunosenescence in MS, SLE, and RA. Notably, cell types that are characteristic of immunosenescence and prone to autoreactivity, i.e., CD4^+^CD28^−^ T cells, ABCs and double negative B cells are expanded in MS. Although their functional features support a possible involvement in MS pathogenesis, it is currently not clear how and to which extent they contribute to the inflammatory processes in the different stages of MS. Hence, they might only reflect the consequences of chronic inflammation rather than the cause of disease. Moreover, the self-generated and self-sustained pro-inflammatory and oxidative environment within the CNS under ongoing recruitment of inflammatory and glial cells, possibly potentiates or causes premature immunosenescence. However, with disease progression the compartmentalized CNS inflammation is also governed by a distinct cellular senescence mechanism. Oxidative stress-induced mitochondrial dysfunction within CNS-resident cells progressively and irreversibly contributes to cellular and continued tissue damage, reduced remyelination capacity, impaired brain plasticity and finally loss of neuro-axonal reserves. Further research is needed to unravel the clinical relevance of these mechanisms, in relationship to immunosenescence, to improve treatments for MS at all ages and disease stages, with an acceptable risk-benefit profile.

## Author Contributions

OP and VvP contributed to the conceptualization of this work and to the research of the current literature on the subject. OP extensively compiled current knowledge on the subject to write and correct the first draft. VvP commented, corrected, and validated the first draft. All authors contributed to the article and approved the submitted version.

## Funding

OP is currently supported by the Fonds de la Recherche Scientifique (F.R.S.-FNRS, Belgium) as a research fellow (Grant No. 40005159).

## Conflict of Interest

The authors declare that the research was conducted in the absence of any commercial or financial relationships that could be construed as a potential conflict of interest.

## Publisher's Note

All claims expressed in this article are solely those of the authors and do not necessarily represent those of their affiliated organizations, or those of the publisher, the editors and the reviewers. Any product that may be evaluated in this article, or claim that may be made by its manufacturer, is not guaranteed or endorsed by the publisher.

## Abbreviations

ABC: age-associated B
AID: autoimmune disease
BBB: blood brain barrier
BCR: B cell receptor
CMV: cytomegalovirus
CNS: central nervous system
CSF: cerebrospinal fluid
DC: dendritic cell
DMT: disease-modifying therapy
EAE: experimental autoimmune encephalomyelitis
EBV: Epstein Barr Virus
FLS: fibroblast-like synoviocyte
HLA-DR: Human Leukocyte Antigen-DR isotype
HSC: hematopoietic stem cell
Ig: immunoglobulin
IFN-I: type I interferon
KREC: K-deleting recombination excision circles
MHC-I/II: major histocompatibility complex of class I or II
miRNA: microRNA
MS: multiple sclerosis
mtDNA: mitochondrial DNA
NET: neutrophil extracellular trap
NK: natural killer
NPC: neural progenitor cell
OPC: oligocendrocyte progenitor cell
PBMC: peripheral blood mononuclear cell
PML: progressive multifocal leukoencephalopathy
PPMS: primary progressive MS
RA: rheumatoid arthritis
RNS: reactive nitrogen species
ROS: reactive oxygen species
RRMS: remitting-relapsing MS
SASP: senescence-associated secretory phenotype
SLE: systemic lupus erythematosus
SPMS: secondary progressive MS
TCR: T cell receptor
Th: T helper cell
TREC: T cell receptor excision circles
Treg: regulatory T cell.
